# Are operative microbiology samples warranted in the setting of a Stage III empyema thoracis?

**DOI:** 10.1186/1749-8090-10-S1-A282

**Published:** 2015-12-16

**Authors:** Naveed Abbas, Michael Tolan, David Healy

**Affiliations:** 1 Department of Cardiothoracic Surgery, St Vincent's University Hospital, Elm Park, Dublin, Republic of Ireland

## Background/Introduction

Surgical management of empyema remains a necessity in the modern era. In many cases the patients are already on anti-microbial therapy prior to referral for definitive surgical management. Surgeons will send samples during the operative case for analysis, but in the setting on ongoing microbiological therapy, these may not prove rewarding.

## Aims/Objectives

We examine the utility of intra-operative microbiology sampling in the setting of thoracic empyema.

## Method

A retrospective review of data for patients undergoing decortication for diagnosed stage III empyema was ascertained via prospectively recorded data. Histopathology and culture records were obtained from January 2011 to December 2014. A total of 67 cases were included. The study focused on intra-operative samples taken for microbiological analysis, cytology and histopathology. Material was obtained from broncho-alveolar-lavage samples, wound swabs, pleural fluid analysis and decorticated material.

## Results

Of the total of 67 patients, 1 was incidentally found to have a mesothelioma. Of the remaining 66, 25.7% (17) patients were found to have positive cultures from cortical samples, however 7 of these patients had no growth from concomitant pleural fluid samples. 74.2% were found to be culture negative. In five cases multiple organisms were cultured. The most common species was Staphylocci spp. in 7 colonies (41.1%), Gram negative rods including Escherichia coli, Pseudomonas spp, Klebsiella in 4 colonies (23.5%), there were 2 patients of Corynebacterium spp and 1 of Aspergillus fumigatus. 2 cases of Mycobacterium tuberculosis, diagnosed on both cultures and confirmed by histopathology, had no growths from pleural fluid.

## Discussion/Conclusion

A significant number of patients had positive cultures. In addition a neoplastic case and TB were identified when not suspected. It remains a recommendation that all patients with surgical decortication should have culture samples and that we must take into account the changing microbiology spectrum when considering antibiotic therapy in earlier stages of empyema thoracis.

